# Ultrasound Imaging with Flexible Array Transducer for Pancreatic Cancer Radiation Therapy

**DOI:** 10.3390/cancers15133294

**Published:** 2023-06-22

**Authors:** Xinyue Huang, Hamed Hooshangnejad, Debarghya China, Ziwei Feng, Junghoon Lee, Muyinatu A. Lediju Bell, Kai Ding

**Affiliations:** 1 Department of Biomedical Engineering, Johns Hopkins University, Baltimore, MD 21287, USAhamed@jhu.edu (H.H.);; 2 Department of Radiation Oncology and Molecular Radiation Sciences, Johns Hopkins University, Baltimore, MD 21287, USA; 3 Department of Electrical and Computer Engineering, Johns Hopkins University, Baltimore, MD 21287, USA

**Keywords:** gastrointestinal malignancies, pancreatic cancer, abdominal motion monitoring, ultrasound imaging, flexible array transducer

## Abstract

**Simple Summary:**

Ultrasound (US) imaging has been widely used for tumor tracking in image-guided radiotherapy. The quality of US images using conventional probes highly depends on user proficiency and anatomical changes, which has severely hindered the use of US-based organ motion monitoring for pancreatic cancer. The flexible array transducer is a novel and promising solution to address the limitation of conventional US probes. At the same time, its strength and flexible geometry also makes the image reconstruction and delay-and-sum (DAS) beamforming very challenging. Inaccuracy in delay calculation results in defocused and distorted US images. In this study, we proposed a novel shape estimation for flexible array transducer to enhance abdomen motion monitoring.

**Abstract:**

Pancreatic cancer with less than 10% 3-year survival rate is one of deadliest cancer types and greatly benefits from enhanced radiotherapy. Organ motion monitoring helps spare the normal tissue from high radiation and, in turn, enables the dose escalation to the target that has been shown to improve the effectiveness of RT by doubling and tripling post-RT survival rate. The flexible array transducer is a novel and promising solution to address the limitation of conventional US probes. We proposed a novel shape estimation for flexible array transducer using two sequential algorithms: (i) an optical tracking-based system that uses the optical markers coordinates attached to the probe at specific positions to estimate the array shape in real-time and (ii) a fully automatic shape optimization algorithm that automatically searches for the optimal array shape that results in the highest quality reconstructed image. We conducted phantom and in vivo experiments to evaluate the estimated array shapes and the accuracy of reconstructed US images. The proposed method reconstructed US images with low full-width-at-half-maximum (FWHM) of the point scatters, correct aspect ratio of the cyst, and high-matching score with the ground truth. Our results demonstrated that the proposed methods reconstruct high-quality ultrasound images with significantly less defocusing and distortion compared with those without any correction. Specifically, the automatic optimization method reduced the array shape estimation error to less than half-wavelength of transmitted wave, resulting in a high-quality reconstructed image.

## 1. Introduction

The organ and tumor movement during radiotherapy (RT) treatment delivery has negative adverse impact on the effectiveness of the RT and its clinical outcome [1]. To compensate for tumor motion and its related uncertainty, as the standard of care, a safety margin is added to the target region. This expansion of irradiated target region results in the surrounding organs at risk (OAR) also getting exposed to radiation dose [2,3]. Moreover, this unwanted OAR irradiation limits the dose escalation. For instance, with less than 10% 3-year survival rate [4], pancreatic cancer is a devastating disease that can greatly benefit from dose escalation. Studies have shown that dose-escalated pancreatic cancer doubles and triples the 2-year and 3-year survival rate [5,6,7,8,9,10,11,12,13]. Real-time motion tracking is the most effective method for motion compensation [2], that in turn, allows RT to be performed with higher precision by adjusting the radiation beam to the tumor location [14] and, thus, enables the dose-escalation [15,16,17,18,19,20,21,22].

The major cause of intrafraction OAR and target motion is respiratory-induced motion. Respiratory-related tumor motion in pancreatic cancer can reach up to 35 mm [23]. This considerably high motion uncertainty can be greatly mitigated by real-time tumor positioning. The tumor positioning methods can be divided into two major techniques: direct and indirect [24]. Direct methods are those that use imaging systems to directly track the tumor position, and such systems are kilovolt X-ray imaging-based methods [25,26,27,28], magnetic resonance (MR) imaging-based approaches [29], electromagnetic sensing systems [30,31], and ultrasound-based techniques [32,33,34,35]. In indirect position tracking technique, the motion trajectory is tracked using a surrogate target that is highly associated with the tumor motion, for instance, thoracoabdominal surface displacement tracking using surface optical trackers. By tracking and analyzing the position of markers that are placed on the skin, external surface motion can be obtained [36,37]. Although, indirect methods are non-invasive, to increase the accuracy these methods, an accurate model is required for converting the motion between the body surface and the internal organ [2,36].

Direct tumor tracking methods are highly accurate, but use of X-ray imaging or electromagnetic sensing can result in exposing patients to extra radiation dose or implanting invasive electromagnetic markers, and the associated cost is high, and they cannot retrofit MR imaging to existing RT treatment machines. On the other hand, ultrasound-based direct approach has several advantages [19,33,38]: (i) low cost, (ii) non-invasive (iii) ability for image enhancement with contrast agents, and (iv) easily integratable with current standard of care. Ultrasound (US) tumor tracking has been successfully applied to prostate intrafraction motion tracking and is commercially available [36,37]. Even though the studies have shown promising results for US-based abdominal intrafraction motion tracking [33,39,40,41], its application in clinics is highly hindered. This is mainly due to the need for a special holder for abdominal sites. The holder should be compatible with treatment setup, and the probe and holder should be placed so that it does not block the radiation [33]. Moreover, the conventional US probes are rigid and operator-dependent [42] and require contact pressure that may cause an anatomical change on each day of radiotherapy delivery and, thus, cause dose deviation from the original treatment plan depending on the original patient anatomy from initial ultrasound probe pressure and location [43,44,45].

The flexible array transducer is a promising design that can address user dependency and induced anatomical changes of the conventional US transducer [46,47,48]. The flexible array transducer is a thin US transducer that can be attached to the body surface and adapt to different geometries [49,50]. In recent years, many studies have shown the application of flexible array transducers in biomedical imaging [50,51]. Our group has been investigating the application of the flexible US probe for real-time tumor tracking during radiotherapy for both abdominal and head-and-neck cancers. Although the flexible probe is wearable and takes the shape of subject’s body, the image reconstruction and ultrasound beamforming are very challenging due to its flexible geometry.

The delay-and-sum (DAS) beamformer is commonly for B-mode image reconstruction from radiofrequency (RF) channel data. DAS requires an accurate array shape to calculate the time-of-flight (ToF) between elements and focal point, and accordingly, apply proper time delays to each channel [52]. As a result, the use of DAS as a flexible array transducer is very challenging as the array shape is generally unknown at any given point in time and changes with the body surface. For a 5 MHz probe, a half-wavelength error in element position, or 0.154 mm, potentially causes the summation of wrongly delayed signals in the opposite phase and introduces signal loss [53]. Therefore, if the array shape is not correctly defined, the reconstructed US image is significant, defocused, and distorted [54].

Numerous approaches have been proposed to address the beamforming problem. De Oliveira et al. [55] and Boerkamp et al. [56] developed flexible array transducers with strain gauges and piezoelectric sensors bonded to their surfaces for detecting local curvature. Although these sensors could estimate curvature with reasonable accuracy, the additional hardware increases costs and limits the number of elements. Chang et al. [57] and Noda et al. [58] proposed mathematical model-based shape estimation algorithms to achieve optimal image quality using flexible array transducers. However, the estimation time for both algorithms is relatively long, resulting in a frame rate that is too low for real-time imaging. Moreover, Huang et al. [59] and Noda et al. [60] developed methods to directly reconstruct B-mode images or estimate array geometry from radio frequency (RF) data of flexible array transducers using deep neural networks (DNNs). Despite their efforts, both methods failed to reduce the array shape error to less than half the wavelength of the transmitted wave and suffered from blurry reconstructed images.

In this study, we propose a novel shape-estimating method for a flexible array transducer using a sequential approach. The optical-based shape-estimation algorithm uses the optical tracking system to collect the spatial coordinates of the array and estimate its shape, while the shape optimization algorithm further optimizes the estimated shape by searching for the array shape that can reconstruct the beamformed image with highest quality without any external device. We conducted phantom and in vivo experiments and evaluated the accuracy of the estimated array shapes and reconstructed ultrasound images. 

## 2. Materials and Methods

### 2.1. Optical-Based Shape Estimation Algorithm

**Figure 1:**
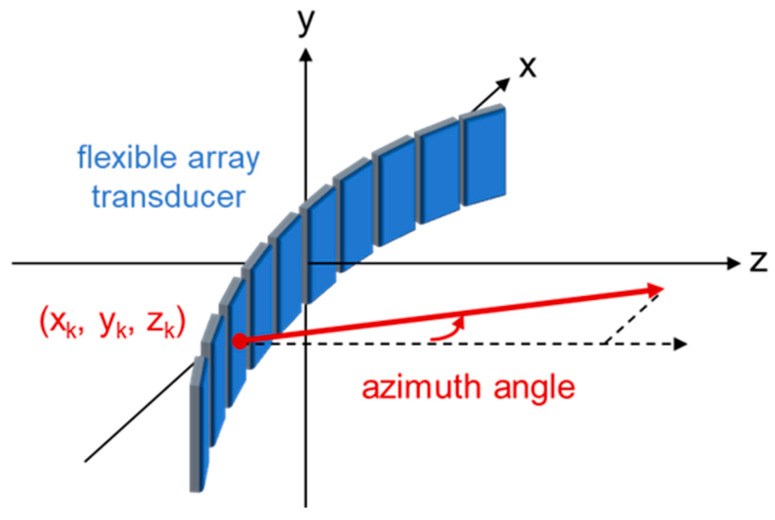
Coordinate system of the flexible array transducer.

The infrared (IR) optical tracker can emit and detect the reflected IR light from the passive marker spheres and triangulate the real-time spatial coordinates of the spheres in 3D space [61]. Several passive sphere markers were fixed on the flexible probe surface and their spatial coordinates were collected. The coordinate system for specifying the flexible array element positions is shown in Figure 1. The center positions of all the array elements are specified with x and z coordinates and azimuth angles (angle from the normal to the positive z axis), while the y coordinates are always equal to 0. However, as the spheres were manually mounted, there would be potential position errors on the y axis. Therefore, we applied the principal component analysis (PCA) to the x-y-z coordinates of the spheres and extracted the first two principal components [62]. The extracted components could be considered as the x and z coordinates of the flexible array.

As the abdominal surface shape of the human is similar to an arc, the shape of the flexible array was first simplified as a concave circular arc with a specific radius. With x-z coordinates of all the spheres, a circle can be fitted to the data points based on the least-squares fitting algorithm developed by Pratt [63], and the radius $R_{fit}$ of this circle is estimated based on the minimization of the following function:

(1) $$R_{fit}=\underset{R_{fit}}{\mathrm{argmin}}\overset{M}{\underset{i=1}{\sum }}\frac{{\left({\left(X_{i}-A\right)}^{2}+{\left(Z_{i}-B\right)}^{2}-R_{fit}^{2}\right)}^{2}}{R_{fit}^{2}}\;$$

where M is the total number of the spheres, $\left(X_{i},Z_{i}\right)$ are the coordinates of the spheres, and (A,B) is the estimated center of the fitted circle. As the optical tracking system measures the coordinates of the spheres’ center, the real radius of the flexible array R can be calculated as follows:

(2) $$R=R_{fit}-R_{sphere}-thickness$$

where $R_{sphere}$ is the radius of the passive marker spheres and thickness is the thickness of the flexible array transducer. Therefore, the center position $\left(x_{k},\;z_{k}\right)$ and the azimuth angle $\alpha_{k}$ of the kth array element can be calculated as follows:

(3) $$x_{k}=R\cdot \sin\left(\frac{2k-K-1}{2}\cdot \frac{p}{R}\right)$$

(4) $$z_{k}=R-R\cdot \cos\left(\frac{2k-K-1}{2}\cdot \frac{p}{R}\right)$$

(5) $$\alpha_{k}=\frac{K+1-2k}{2}\cdot \frac{p}{R}$$

where K is the total element number, p is the pitch, and the center of the array was set at (0, 0). In this way, the array shape could be fully described by $x_{k}$, $z_{k}$, and $\alpha_{k}$.

### 2.2. DAS Beamforming

With the knowledge of the array shape, the DAS beamforming can be performed to reconstruct the beamformed image. Specifically, the time-of-flight or $\tau_{ToF}$ from the tth transmitting element to the rth receiving element through the focal point $\left(x_{f},\;z_{f}\right)$ is formulated as follows:

(6) $$\tau_{ToF}\left(t,r,x_{f},z_{f}\right)=\frac{\sqrt{{\left(x_{f}-x_{t}\right)}^{2}+{\left(z_{f}-z_{t}\right)}^{2}}+\sqrt{{\left(x_{f}-x_{r}\right)}^{2}+{\left(z_{f}-z_{r}\right)}^{2}}}{c}$$

where c is a constant speed of sound. Then, the RF channel data can be properly delayed and summed. The focal point in the beamformed image $I\left(x_{f},\;z_{f}\right)$ is reconstructed as follows:

(7) $$I\left(x_{f},\;z_{f}\right)=\overset{T}{\underset{t=1}{\sum }}\overset{R}{\underset{r=1}{\sum }}\overset{N}{\underset{n=1}{\sum }}RF\left(t,r,n\right)\cdot \delta_{t,r}\left(n\right)$$

where *T* is the number of transmitting elements, R is the number of receiving elements, N is the length of receiving samples, RF(t,r,n) represents the tth transmitting elements, rth receiving elements, and nth sample of the RF channel data. $\delta_{t,r}\left(n\right)$ is the Kronecker delta function for data extraction expressed as follows [58]:

(8) $$\delta_{t,r}\left(n\right)=\{\begin{array}{c} 1,\;\;if\;\left|n-\tau_{ToF}\left(t,r,x_{f},z_{f}\right)\cdot f_{s}\right|\leq \frac{1}{2}\\ 0,\;\;if\;\left|n-\tau_{ToF}\left(t,r,x_{f},z_{f}\right)\cdot f_{s}\right|>\frac{1}{2} \end{array}$$

where $f_{s}$ is the sampling frequency. In this way, the beamformed image is reconstructed from the RF channel data based on the estimated array shape.

### 2.3. Shape Optimization Algorithm

As mentioned, a half-wavelength error in element position will result in significant defocusing in the reconstructed image. However, in clinical practice, the spatial resolution of the optical tracking system is normally larger than half-wavelength of the transmitted wave from the flexible array transducer (e.g., 0.25 mm accuracy with the NDI Polaris Spectra System). Therefore, the optical-based shape estimation algorithm may not achieve the expected accuracy of array shape. To further improve the accuracy, we developed a shape optimization algorithm that could describe the array shape with a more detailed model and search for the optimal shape to reconstruct ultrasound images with the highest quality.

#### 2.3.1. Array Shape Model

A more detailed mathematical model was designed to describe the flexible array shape. As the center-to-center distance between two successive elements is fixed as the pitch p, the array shape could be fully described with K−1 parameters $P=\left\{\Delta \alpha_{1},\Delta \alpha_{2},\ldots ,\Delta \alpha_{K-1}\right\}$, where $\Delta \alpha_{k}$ is the external angle between the kth element and the k+1th element. Therefore, the azimuth angle $\alpha_{k}$ of the kth array element can be calculated as follows:

(9) $$\alpha_{k}=\{\begin{array}{cc} 0,\; & if\;k=1\\ \overset{k-1}{\underset{i=1}{\sum }}\Delta \alpha_{i}, & \;\;\;\;\;\;\;\;if\;1<k\leq K \end{array}$$

With the knowledge of azimuth angles $\left\{\alpha_{1},\alpha_{2},\ldots ,\alpha_{K}\right\}$ and pitch p, the center position $\left(x_{k},\;z_{k}\right)$ of the kth array element can be calculated as follows:

(10) $$x_{k}=\{\begin{array}{cc} 0, & if\;k=1\\ p\cdot \overset{k-1}{\underset{i=1}{\sum }}\cos\alpha_{i}, & \;\;\;\;\;\;\;\;if\;1<k\leq K \end{array}$$

(11) $$z_{k}=\{\begin{array}{cc} 0, & if\;k=1\\ p\cdot \overset{k-1}{\underset{i=1}{\sum }}\sin\alpha_{i}, & \;\;\;\;\;\;\;\;\;if\;1<k\leq K \end{array}$$

where the azimuth angle of the first element is set to 0, and the center position of the first element is set at (0,0). Then, the DAS beamforming can be performed to reconstruct the beamformed image I from the RF channel data based on this array shape model.

#### 2.3.2. Evaluation of Beamformed Image

An evaluation method for the beamformed image I was selected to indicate the accuracy of the estimated array shape. Inspired by the maximum entropy (MEM) image reconstruction algorithm [64], studies on transducer array shape estimation [58], and autofocusing of synthetic-aperture radar (SAR) images [65,66], we used the Shannon image entropy as the metric for evaluating the quality of beamformed images [67]. The beamformed image I was normalized to $I_{norm}$, and its entropy H can be calculated with the following function:

(12) $$H\left(I_{norm}\right)=-\overset{M}{\underset{i=1}{\sum }}p_{i}\log_{2}p_{i}$$

where $p_{i}$ represents the probability of seeing the ith possible outcome of the beamformed image $I_{norm}$. Entropy measures the information contents of images, and entropies near 0 indicate images with little or no information, while larger entropies indicate more information contents. However, there is a conflict between different studies. MEM maximizes the entropy to optimize the reconstructed image, while SAR autofocusing minimizes the entropy to achieve best focus. To correctly use the entropy for our study, we tested the relationship between the array shape accuracy and the beamformed image entropy.

**Figure 2:**
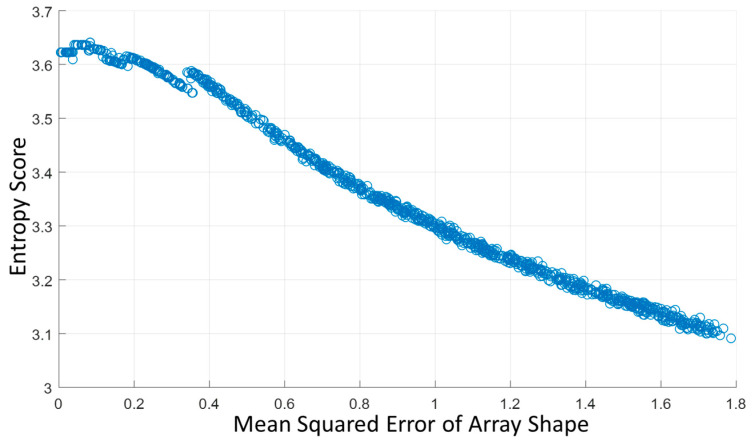
Scatter plot of mean absolute errors of the array shape and entropy scores of the corresponding reconstructed images from 1000 random shape assumptions.

When performing the test scans on different imaging targets, the flexible array transducer was set in a flat shape to make the ground truth of the array shape known. Then, different array shape assumptions were randomly generated and used for reconstructing the beamformed image. The mean absolute errors between the assumptions and ground truth were plotted versus the entropy of their corresponding beamformed images. Figure 2 shows an example of the scatter plot of mean absolute errors of the array shape and entropy scores of the corresponding reconstructed images from 1000 random shape assumptions.

The results show that the entropy of the beamformed image tends to be larger when the array shape error is smaller. Therefore, we set the principle that the beamformed image using an array shape of higher accuracy would have a larger entropy. More in-depth discussions about the effect of entropy in ultrasound images will be presented in the discussion.

#### 2.3.3. Array Shape Optimization

**Figure 3:**
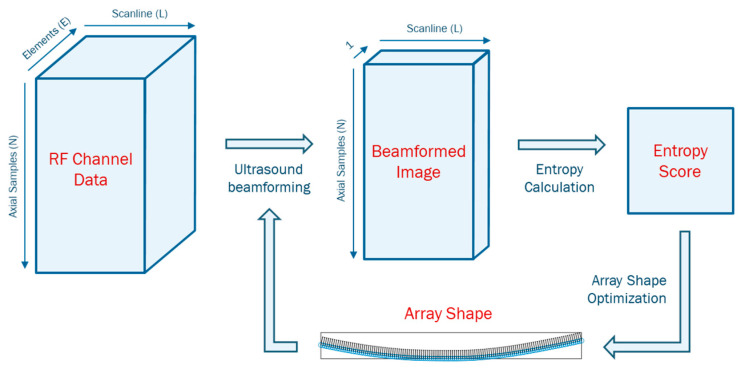
An overview of the proposed shape optimization algorithm.

The circular arc array shape estimated using the optical-based shape estimation algorithm is used as the initial shape assumption for further optimization. The optimal shape parameters $P=\left\{\Delta \alpha_{1},\Delta \alpha_{2},\ldots ,\Delta \alpha_{K-1}\right\}$ are globally searched to achieve maximum entropy of the corresponding beamformed image. The following global maximum problem was solved:

(13) $$\hat{P}\;=\;\underset{P}{\mathrm{argmax}}f(P),\;\;\;\;lb\;\leq \;P\;\leq \;ub$$

where f(P) is the entropy evaluation function of the beamformed image reconstructed based on P, and lb and ub are the lower bounds and upper bounds of the shape parameters. The optimization is terminated when the stopping criterion is met. Overview of the shape optimization algorithm is presented in Figure 3. Details on the optimization model, constraints, and conditions are described in the methods.

### 2.4. Scan Conversion

**Figure 4:**
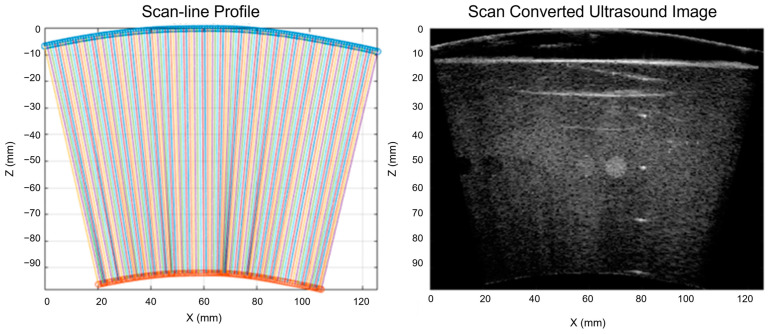
Profile of each scan-line and the corresponding scan converted ultrasound image.

With the knowledge of azimuth angle and center position of each element, the angle and origin position of each transmission scan-line can be determined. Scan conversion is performed to transform the polar coordinate ultrasound data into Cartesian coordinate data. Bilinear interpolation is applied to the scan conversion task. The method uses four adjacent data points in the beamformed image $\left\{I(x_{l},z_{l}),I(x_{l},z_{u}),I(x_{u},z_{l}),I(x_{u},z_{u})\right\}$ to compute the value for the needed pixel S(x,z), which can be expressed as follows [68,69]:

(14) $$T_{1}=\frac{x_{u}-x}{x_{u}-x_{l}}\cdot I\left(x_{l},z_{l}\right)+\frac{x-x_{l}}{x_{u}-x_{l}}\cdot I\left(x_{u},z_{l}\right)$$

(15) $$T_{2}=\frac{x_{u}-x}{x_{u}-x_{l}}\cdot I\left(x_{l},z_{u}\right)+\frac{x-x_{l}}{x_{u}-x_{l}}\cdot I\left(x_{u},z_{u}\right)$$

(16) $$S\left(x,z\right)=\frac{z_{u}-x}{z_{u}-z_{l}}\cdot T_{1}+\frac{z-z_{l}}{z_{u}-z_{l}}\cdot T_{2}$$

where $T_{1}$ and $T_{2}$ are the intermediate points for computing the pixel value S(x,z). Then, the imaging region is calculated based on the scan-line profile, and a binary mask is applied to the interpolated image to get the scan converted ultrasound image. Figure 4 illustrates the profile of each scan-line and the corresponding scan converted ultrasound image.

### 2.5. Experiments

#### 2.5.1. Optical-Based Shape Estimation Experiments

The NDI Polaris Spectra System (Northern Digital Inc., Waterloo, ON, Canada), rigid body tools, and passive marker spheres were all used for optical-based shape estimation. Five passive marker spheres were attached to the back surface of a flexible array transducer (made by Hitachi and Japan Probe, Yokohama, Japan). Parameters of the transducer are listed in Table 1. All the spheres were aligned with the transducer array, and the first and last spheres were positioned at the two ends of the transducer array. The spatial coordinates of all the spheres were collected using the optical tracker, and the proposed shape estimation algorithm was implemented in the MATLAB (version R2020b, MathWorks, Natick, MA, USA) to estimate the array shape.

**Figure 5:**
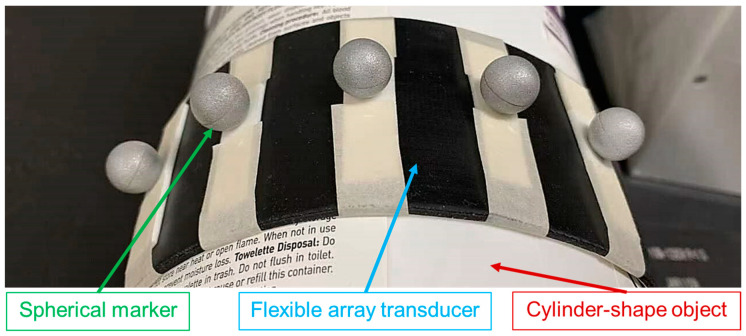
Experimental set-up for evaluating the array shape.

Three cylinder-shaped objects with known radius $R_{object}$ were used to evaluate the accuracy of the shape estimation algorithm. The experimental set-up is shown in Figure 5. The flexible array transducer was attached to the surface of the cylinder-shaped object, and the radius of the transducer array $R_{array}$ was estimated with the proposed algorithm.

The flexible array transducer was also attached to the surface of the abdominal region of an ABDFAN Abdominal Ultrasound Phantom (Kyoto Kagaku Co., Kyoto, Japan) in lateral direction. The cross-sectional X-ray image of the phantom and transducer was captured using a Cios Alpha C-arm imaging system (Siemens Healthineers, Erlangen, Germany). Then, the transducer array was segmented from the X-ray image and fitted into a curve shape. This segmented array shape was considered as the ground truth and compared with the optical-based estimated and optimized array shape.

#### 2.5.2. RF Data Acquisition and Image Reconstruction

**Figure 6:**
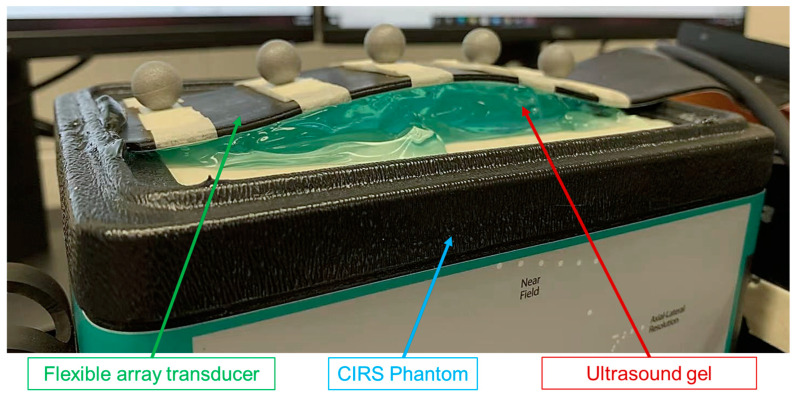
Experimental set-up for the CIRS phantom scan.

The flexible array transducer connected with the Vantage System (Verasonics Inc., Kirkland, WA, USA) was used to scan a CIRS General Purpose Ultrasound Phantom (Computerized Imaging Reference Systems Inc., Norfold, VA, USA), liver area of an ABDFAN Abdominal Ultrasound Phantom (Kyoto Kagaku Co., Japan), and a healthy volunteer under Johns Hopkins Institutional Review Board approval. Ultrasound gel was used to create a random curvature for the scanning surface of flexible array transducer, which is depicted in Figure 6. In addition, a linear array transducer (ATL L7-4 38 mm, Philips Healthcare, Cambridge, MA, USA) was used to scan the same imaging region. Parameters of the linear array transducer is shown in Table 2, and the speed of sound was set as uniform at 1540 m/s. The RF channel data was acquired by transmitting acoustic signals and receiving the backscattered signals.

The DAS beamforming, scan conversion, and image post-processing were implemented in MATLAB. The RF channel data and estimated array shape parameters were used to reconstruct the “corrected” ultrasound images. In addition, the RF channel data was wrongly delayed and reconstructed under the assumption that the flexible array is flat and linear. These “uncorrected” results stand for the images reconstructed without any correction on array shape. The RF channel data from the linear array transducer was also reconstructed as the ground truth, which will be evaluated and compared with the “corrected” and “uncorrected” results of the flexible array transducer.

#### 2.5.3. Shape Optimization Algorithm Implementation

The shape optimization algorithm was implemented in the MATLAB with the “surrogateopt” function, which is a global solver for time-consuming objective functions. To achieve a faster convergence, the initial shape parameters $P_{0}$ were set to the circular arc array shape estimated by optical-based estimation algorithm. In addition, to keep the array shape smooth and continuous, lower bounds lb and upper bounds ub were set as follows:

(17) $$lb=P_{0}-\pi /720,\;\;ub=P_{0}+\pi /720$$

The bounding values were set based on the abdominal surface shape of the human. The shape optimization was terminated after 200 iterations. The array shape parameters $P=\left\{\Delta \alpha_{1},\Delta \alpha_{2},\ldots ,\Delta \alpha_{K-1}\right\}$ were optimized to achieve maximum entropy of the beamformed image.

### 2.6. Evaluation Metrics

As defocusing and distortion are the two major problems of the reconstructed ultrasound image without the knowledge of array shape, the following metrics were used to evaluate the quality and accuracy of the results. For the CIRS phantom results, the defocusing effect was assessed by evaluating the lateral full-width-at-half-maximum (FWHM) of all the point scatters, while the image distortion was evaluated by the aspect ratio of the cyst. The aspect ratio is the ratio of lateral and axial axes of the cyst. An ellipse with aspect ratio of 1 is a circle:

(18) $$Aspect\;Ratio=\frac{Lateral\;Axis}{Axial\;Axis}$$

For the ABDFAN phantom and liver scan results, the cysts, blood vessels, and muscles were manually segmented. The image distortion was evaluated by computing the Sørensen–Dice coefficient (Dice score) [70], Jaccard similarity coefficient (Jaccard index) [71], and Hausdorff distance [72] between the segmentations of flexible array transducer results and the ground truth (linear array transducer results). Before the evaluation, rigid registration was performed to the segmentations to eliminate the translational and rotational difference between the results and ground truth. For X and Y being two non-empty sets of the segmentation, the Dice score DSC, Jaccard index J, and Hausdorff distance HD can be computed as follows:

(19) $$DSC=\frac{2\left|X\cap Y\right|}{\left|X\right|+\left|Y\right|},\;\;J=\frac{\left|X\cap Y\right|}{\left|X\cup Y\right|}$$

(20) $$HD\left(X,Y\right)=\underset{x\in X}{\max}\left\{\;\underset{y\in Y}{\min}\left\{\;d\left(x,y\right)\;\right\}\;\right\}$$

where x and y are points in the sets X and Y, respectively, and d(x,y) is the Euclidian distance between points x and y. In addition, the contrast-to-noise ratio (CNR) and generalized CNR (GCNR) [73] were used to evaluate the quality of the reconstructed image. The CNR and GCNR are calculated from the inner and outer regions defined in the figures:

(21) $$CNR=20\;log_{10}\left(\frac{\left|\mu_{out}-\mu_{in}\right|}{\sqrt{\sigma_{out}^{2}+\sigma_{in}^{2}}}\right)\;,$$

where $\mu_{in}$ and $\mu_{out}$ are the mean intensity, and $\sigma_{in}$ and $\sigma_{out}$ are the standard deviation of the inner and outer region, respectively. The GCNR is defined as:

(22) $$GCNR=1-\int \min\left\{p_{out}\left(x\right),p_{in}\left(x\right)\right\}dx.$$

where $p_{in}\left(x\right)$ and $p_{out}\left(x\right)$ are the probability density function of the signal of inner and outer region, and x is the pixel intensity. If GCNR=0, the distributions are entirely overlapped; if *GCNR* = 1, the distributions are completely independent, which represents a high contrast.

## 3. Results

### 3.1. Array Shape Estimation and Optimization

**Figure 7:**
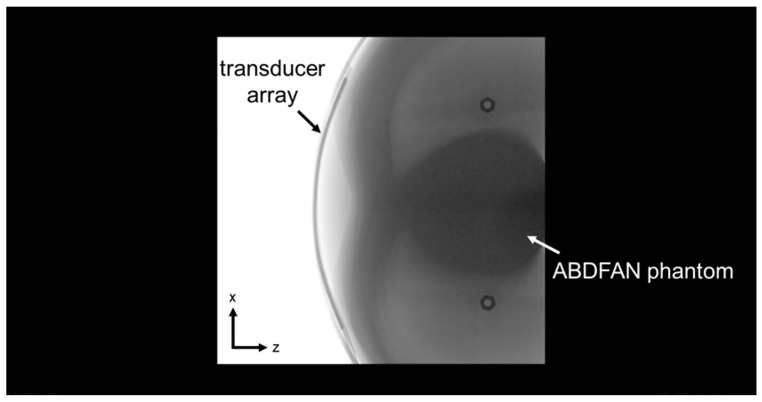
X-ray image of the flexible array transducer with the ABDFAN phantom.

**Figure 8:**
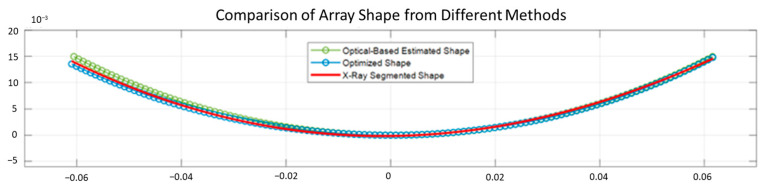
Comparison of the estimated array shapes from different methods.

The ground truth radius of the cylinder-shaped objects $R_{object}$ and the estimated radius of the flexible array $R_{array}$ from the optical-based shape estimation algorithm are listed in Table 3. The absolute errors of the radii are 0.20 mm for object 1, 0.73 mm for object 2, and 1.55 mm for object 3. The cross-sectional X-ray image of the ABDFAN phantom and the flexible array transducer is shown in Figure 7, and the black line on the surface of the phantom is the array. Figure 8 depicts the comparison of the array shape results from different methods, where the X-ray segmented shape (red line) is considered as the ground truth. The mean absolute error of the element positions between the ground truth and optical-based estimated shape is 0.3604 mm, while that of the optimized shape is 0.1488 mm. Half-wavelength of the transmitted wave is 0.15 mm, and therefore, the shape optimization algorithm could achieve the accuracy for reconstructing optimal ultrasound images.

### 3.2. CIRS Phantom Results

**Figure 9:**
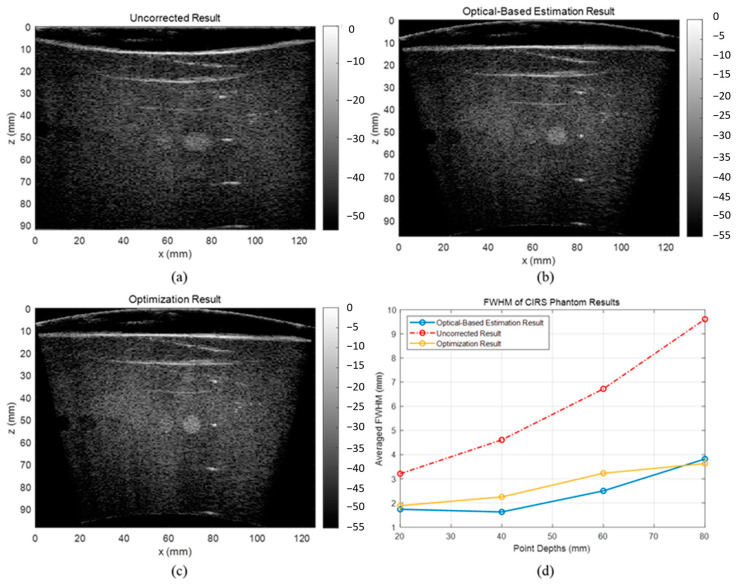
Reconstructed images of the CIRS phantom (**a**) without array shape correction, (**b**) with optical-based estimated shape, and (**c**) with optimized shape, and (**d**) line plot of lateral FWHM of the point scatters.

The uncorrected, optical-based estimation, and optimization results of the CIRS phantom are shown in Figure 9. The results illustrate that without any correction on array shape, the reconstructed image will have strong defocusing and distortion, while both proposed algorithms can correct these effects. The lateral FWHM of the point scatters in different depths are plotted in Figure 9d. The averaged lateral FWHM of the uncorrected, estimation, and optimization results are 6.04 mm, 2.42 mm, and 2.75 mm, respectively. The aspect ratio, CNR, and GCNR of the center hyperechoic cyst and the second left anechoic cyst are listed in Table 4. Both corrected results have significantly lower distortion and higher contrast than the uncorrected result. Specifically, analyzing the cysts in the CIRS phantom, the optimization result has a more accurate shape, clearer boundary, and higher contrast compared with the optical-based estimation result. Therefore, it is believed that the shape optimization algorithm has the overall best performance on estimating the array shape.

### 3.3. ABDFAN Phantom and Liver Scan Results

**Figure 10:**
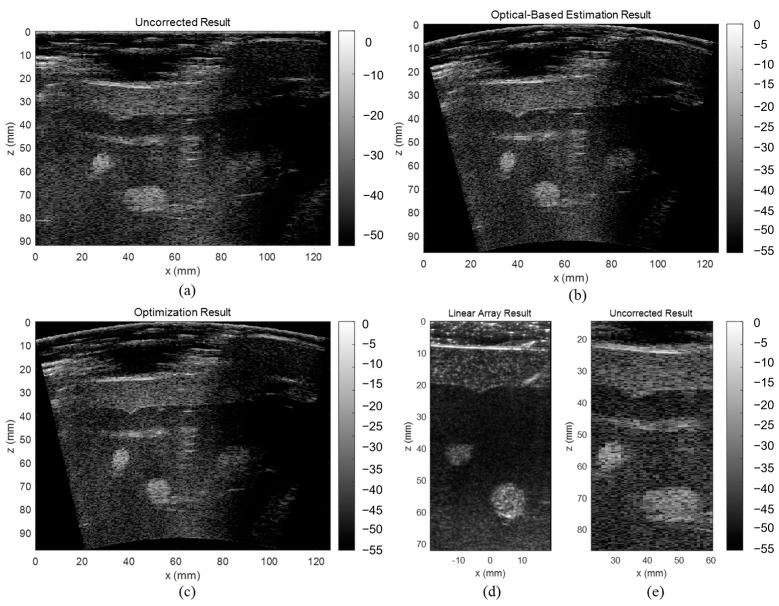
Reconstructed images of the ABDFAN phantom (**a**) without array shape correction, (**b**) with optical-based estimated shape and (**c**) optimized shape, (**d**) ground truth image from the linear array transducer, and (**e**) cropped image with the same region as the ground truth.

**Figure 11:**
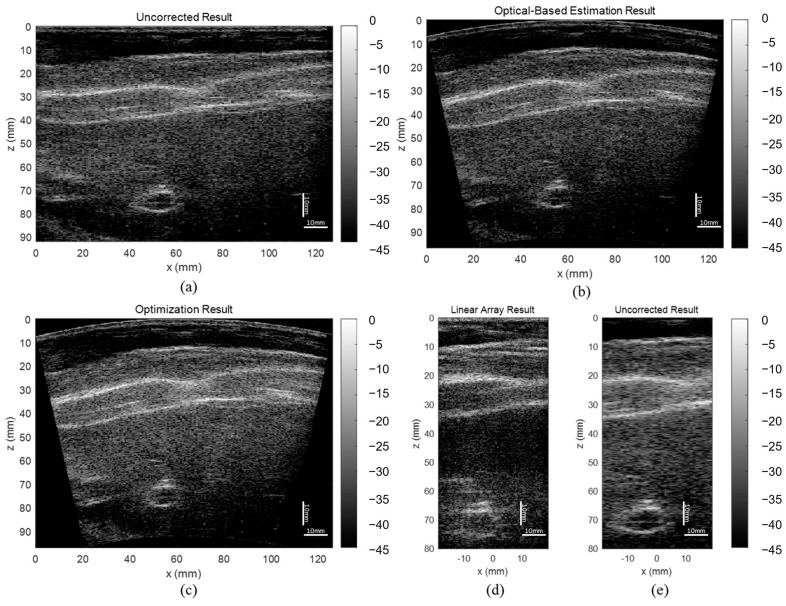
Reconstructed images of the liver scan (**a**) without array shape correction, (**b**) with optical-based estimated shape and (**c**) optimized shape, (**d**) ground truth image from the linear array transducer, and (**e**) cropped image with the same region as the ground truth.

**Figure 12:**
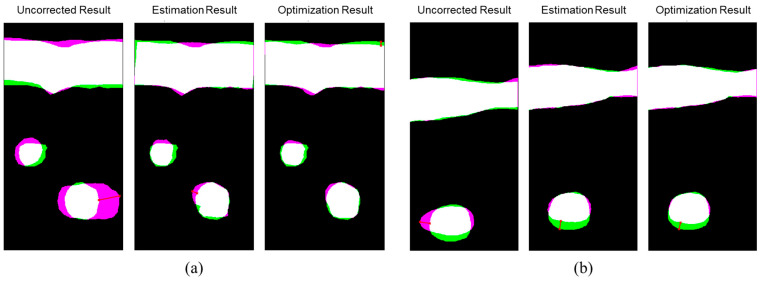
Comparison of the segmentations between the flexible array transducer results and the ground truth for the (**a**) ABDFAN phantom and (**b**) liver scan. Green regions represent the ground truth, pink regions represent the reconstructed results, white regions represent the common regions, and the red arrows represent the Hausdorff distances.

The uncorrected, optical-based estimation, and optimization results of the ABDFAN phantom and liver scan are shown in Figure 10 and Figure 11. The ground truth images from the linear array transducer are shown in Figure 10d and Figure 11d, and the same regions are cropped from the flexible array transducer results, and examples of the uncorrected results are depicted in Figure 10e and Figure 11e. The uncorrected results have significant distortions compared with the ground truth and corrected results. To quantitatively analyze the distortion, the cysts, blood vessels, and mussels are segmented as shown in Figure 12, and the Dice score, Jaccard index, and Hausdorff distance between the results and ground truth are evaluated and listed in Table 5, Table 6, and Table 7. The results show that both estimation and optimization algorithms can correct the distortions of the reconstructed image, and there is no significant difference between the two algorithms. The CNR and GCNR of the center cyst in the ABDFAN phantom and the large blood vessel in the liver scan are listed in Table 5, Table 6, and Table 7. In conclusion, the images reconstructed by both algorithms have an overall higher accuracy and contrast than the uncorrected images, and the optimization algorithm has a slightly better performance on estimating the array shape.

## 4. Discussion

The evaluation results showed that the proposed optical-based shape estimation algorithm could successfully estimate the array shape of the flexible array transducer with reasonable accuracy and reconstruct ultrasound images with significantly less defocusing and distortion than those without any shape correction. The proposed shape optimization algorithm could further improve the accuracy of the array shape estimation. The mean absolute error of the element positions was less than half-wavelength of the transmitted wave, which means the algorithm could reconstruct ultrasound images with optimal accuracy and quality. In addition, the computation time of the shape estimation algorithm was less than 0.01 s, while that of the shape optimization algorithm for 200 iterations was about 1000 s. Therefore, the proposed shape estimation algorithm could achieve real-time ultrasound imaging with acceptable accuracy and frame rate. For specific frames that require higher accuracy and quality, the shape optimization algorithm could be used to further improve the reconstructed images.

The Shannon entropy is a measurement of information and uncertainty in random variables [67]. Hughes first used the entropy for analyzing ultrasound signals and indicated that entropy can be used to quantitatively characterize the changes in the microstructures of scattering media [74,75]. Tsui et al. applied the entropy of ultrasound backscattered signals to multiple diseases assessment [76,77]. The studies concluded that increasing the scatterer concentration would generate a stronger effect of constructive wave interference and lead to a larger backscattered amplitude. In this condition, various echo amplitudes exist, and the signal uncertainty and unpredictability (entropy) increase. Noda et al. proposed an assumption that the beam-summed image using an array shape of higher accuracy would have smaller entropy [58]. However, there was no in-depth discussion of this assumption. To the best of our knowledge, there is no study on evaluating the ultrasound beamforming process with entropy.

**Figure 13:**
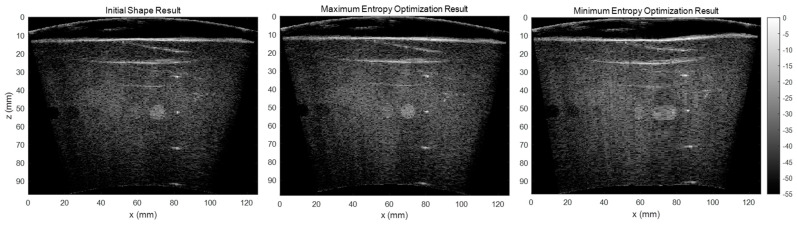
Reconstructed images with the initial shape, the maximum entropy-optimized shape, and the minimum entropy-optimized shape.

As the RF channel data is delayed and summed based on the array shape to form the beamformed image, errors in array shape will make the delayed RF data sum in the opposite phase, which causes significant signal loss. It would lead to a smaller backscattered amplitude and the signal’s entropy would increase. In addition to our testing of the correlation between the array shape accuracy and beamformed image entropy, we also tested the effect of entropy by maximizing and minimizing the entropy in our shape optimization algorithm and compared the results. Figure 13 shows the reconstructed images using the initial shape, maximum entropy-optimized shape, and minimum entropy-optimized shape. The results illustrate that the array shape optimized by minimizing the entropy is worse than the initial shape. Therefore, we believe that the beamformed ultrasound image using an array shape of higher accuracy would have a larger entropy.

Here, we presented a shape estimation approach that consists of two sequential methods to estimate the array shape and reconstruct B-mode images acquired by flexible array transducer using deep learning method. One potential application of our method is to improve the ultrasound imaging reconstruction technique for in vivo tumors shape and size calculations [78]. With current developments of nanomedicine, it has shown a great potential for diagnosis and treatment of many disease [79]. Nanocarriers can actively and precisely target the tumor by binding to the cancer cell-overexpressed receptors [80]. However, tumor size and shape might be an important consideration for designing nanocarriers [79]. The method presented in this paper may increase the accuracy of tumor shape and size estimation, which in turn, can increase the efficiency of nanocarriers design. Future studies are warranted to demonstrate the effectiveness of our method for shape estimation.

There are some limitations of this study: First, the computation time of the shape optimization algorithm is long. As it is explained in the methods, the Kronecker delta function $\delta_{t,r}\left(n\right)$ was used for reconstructing the beamformed image I. As $\delta_{t,r}\left(n\right)$ cannot be differentiated by the shape parameters P, our shape optimization algorithm was implemented with a global solver without using the gradient to P, which significantly increased the computation time compared with gradient decent based optimizer. Inspired by the previous literature [58], a future direction of this study will be replacing the Kronecker delta function with a function that is differentiable by P. Second, the optical-based estimation algorithm relies on external devices including the infrared optical tracker and passive marker spheres, which may be occluded during radiotherapy. The optical tracking system should be properly set up in advance to avoid any interference. In addition, the speed of sound was set to be homogeneous in this study. Changes in the speed of sound will influence the ToF of the focal points, which has the same effect as the errors in array shape. Therefore, the proposed shape optimization algorithm may simultaneously correct the array shape and the heterogeneous speed of sound. Future work needs to be conducted to study how heterogeneous speed of sound may affect the performance of the proposed algorithms.

## 5. Conclusions

In this study, we proposed a novel shape estimation approach that consists of two sequential methods to estimate the array shape and reconstruct B-mode images acquired by flexible array transducer. The optical-based shape estimation algorithm used the optical tracking system to collect the spatial coordinates of the array and estimate the array shape as a circular arc with specific radius. The shape optimization algorithm searched for the array shape that maximized the entropy of its beamformed image. The evaluation results showed that the estimation algorithm could reconstruct ultrasound images with significantly less defocusing and distortion than those without any correction, while the optimization algorithm could further reduce the array shape error to less than half-wavelength of the transmitted wave and improve the accuracy and quality of the reconstructed images. Therefore, the proposed algorithms have the potential to enable high-quality real-time ultrasound imaging with the flexible array transducer. Additionally, as no holders are required for probe fixation, the real-time reconstitution enables US-based gastrointestinal organ motion monitoring during the radiotherapy.

## Figures and Tables

**Table 1:** Flexible Array Transducer Parameters.

Parameters	Value
Number of Elements	128
Center Frequency	5 MHz
Element Width	0.8 mm
Element Pitch	1.0 mm
Element Length	10 mm
Thickness	1.5 mm
TX Elements	64
RX Elements	128

**Table 2:** Linear Array Transducer Parameters.

Parameters	Value
Transducer Model	ATL L7-4
Number of Elements	128
Center Frequency	5 MHz
Element Pitch	0.298 mm
Element Length	7 mm
TX Elements	64
RX Elements	128

**Table 3:** Comparison of the Ground Truth and the Optical-based Estimated Radius.

	$R_{\mathrm{object}}$	$R_{\mathrm{array}}$
Object 1	110.00 mm	110.20 mm
Object 2	72.50 mm	71.77 mm
Object 3	65.00 mm	63.45 mm

**Table 4:** Evaluation Results of the Hyperechoic Cyst in the CIRS Phantom.

	Aspect Ratio	CNR	GCNR
Uncorrected	1.68	1.26 dB	0.62
Optical-based estimation	1.08	3.69 dB	0.77
Optimization	1.00	4.44 dB	0.81

**Table 5:** Evaluation Results of the Anechoic Cyst in the CIRS Phantom.

	Aspect Ratio	CNR	GCNR
Uncorrected	1.53	−8.51 dB	0.28
Optical-based estimation	1.08	−8.51 dB	0.26
Optimization	1.03	−6.92 dB	0.29

**Table 6:** Evaluation Results of the ABDFAN Phantom.

	Dice	Jaccard	Hausdorff	CNR	GCNR
Uncorrected	0.87	0.77	6.73 mm	2.78 dB	0.71
Estimation	0.95	0.90	1.66 mm	2.92 dB	0.75
Optimization	0.95	0.91	1.50 mm	3.86 dB	0.78

**Table 7:** Evaluation Results of the Liver Scan.

	Dice	Jaccard	Hausdorff	CNR	GCNR
Uncorrected	0.93	0.87	3.49 mm	−13.11 dB	0.18
Estimation	0.95	0.90	2.92 mm	−11.08 dB	0.20
Optimization	0.96	0.92	2.40 mm	−9.83 dB	0.22

## Data Availability

The raw data supporting the conclusions of this article will be made available by the authors upon request.
